# Letter to the editor: health professionals’ attitudes toward individuals with eating disorders: who do we think they are?

**DOI:** 10.1186/s40337-017-0150-6

**Published:** 2017-07-17

**Authors:** Deborah Lynn Reas, Kjersti Solhaug Gulliksen, Johanna Levallius, Rasmus Isomaa

**Affiliations:** 1 Regional Department for Eating Disorders, Division of Mental Health and Addiction, Oslo University Hospital, P.O. Box 4956, Nydalen, N-0424 Oslo, Norway; 2 Department of Psychology, Faculty of Social Sciences, University of Oslo, Oslo, Norway; 3 Department of Eating Disorders Gaustad, Division of Mental Health and Addiction, Oslo University Hospital, Oslo, Norway; 4 Resource Center for Eating Disorders, Department of Clinical Neuroscience, Karolinska Institute, Stockholm, Sweden; 5 Department of Social Services and Health Care, City of Jakobstad, Finland

**Keywords:** Stigma, Health professionals, Attitudes, Beliefs, Stereotypes, Eating disorders literacy, DSM-5

## Abstract

Health professionals are not immune to stigmatizing attitudes and stereotypes found in society-at-large. Along with patients and their loved ones, treatment providers are important stakeholders – and gatekeepers – in the successful delivery of mental healthcare. Prevailing attitudes among professionals can facilitate timely recognition, enable access to care and uptake of evidence-based practices, or undermine help-seeking and therapeutic engagement. At an interactive activity at the 2016 Nordic Eating Disorders Society (NEDS) meeting, we asked health professionals to describe individuals with eating disorders. The most common descriptive term used was “anxiety” followed by “thin”, “sad”, “control”, "female", and "suffering/pain". Further research on professionals’ attitudes toward individuals with eating disorders is necessary to inform education, awareness, and advocacy efforts following the diagnostic revisions in the DSM-5.

## Background

Health professionals play a vital role in connecting science to service, and bridging bench-to-bedside gaps in the delivery of care, yet they are not immune to lay stereotypes or stigmatizing beliefs found in the community [1]. Individuals with eating disorders (ED) have been viewed by society-at-large as attention-seeking, blameworthy, or as having a trivial, self-imposed problem [2], and viewed by professionals as vain, manipulative, or difficult [3, 4]. These findings are particularly worrying in light of studies of patient perspectives on treatment-seeking and engagement in ED. Individuals with ED highly value clinician attributes such as acceptance, empathy, warmth, and openness, whereas negative clinical encounters are characterized by a judgmental stance, disregard, or prejudice by health professionals [5]. Frequency of stigma exposure is associated with numerous adverse effects on health and well-being for those with ED, including greater ED symptomology, depression, and lower self-esteem [6]. Perceived stigma, or fear thereof, is consistently recognized as a prominent barrier to help-seeking for ED [7], diminishing our ability to identify and effectively treat all who may benefit [8].

Traditional views that ED are afflictions of “thin, affluent, young, white women” [9], render higher-weight individuals, older individuals, males, and ethnic minorities highly susceptible to bias and under-detection. Symptoms may go unrecognized, misinterpreted, or dismissed due to health professionals’ expectations about the presentation of an ED. The DSM-5 criteria for ED have recently undergone changes with the removal of female-centric criteria (i.e., amenorrhea) and pejorative terminology (i.e., “refusal” to maintain weight). How these diagnostic changes might affect provider attitudes toward individuals with ED is unclear. More research is also needed to understand professionals’ attitudes toward newly added diagnostic labels, including *avoidant-restrictive food intake disorder* and *binge eating disorder*, as well as atypical presentations such as muscle dysmorphia [10].

### Putting it into words: who do we think they are?

**Fig. 1:**
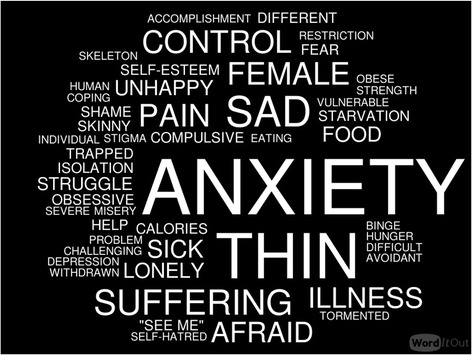
In a word, how do professionals describe individuals with ED? The typeface is scaled proportional to the frequency of each term. The figure was generated using https://worditout.com

An interactive activity at the 2016 Nordic Eating Disorders Society (NEDS) in Helsinki, Finland offered a recent glimpse into professionals’ views toward individuals with ED. The main conference theme of the 2016 meeting was “*Information and Misinformation,*” and 3 days were organized to highlight common myths and misconceptions of ED [9]. At one of the plenaries, the audience was instructed to write down the “first word that comes to mind” to describe someone with an ED. Over 150 professionals attended, with 6 months to 35 years of experience in the field of ED. Limitations notwithstanding, this activity provided a rapid assessment of attitudes and associations at a manifest level and offers an interesting, if not powerful, visual (see Fig. 1). Many words reflected the profound and devastating toll of an ED (e.g., *suffering, pain, trapped, struggle*). Responses specific to ED pathology (e.g*., food*) were less common than associated features or comorbidity. Overall, *anxiety* was the most frequent response, followed *by thin, sad, control,* female, and suffering/pain.

## Conclusions

Whether thinness and female-centric words reflect lingering stereotypes of ED, or simply reflect the clientele treated by this group of professionals is unclear, yet findings deserve further investigation given the implications for potential bias and ascertainment. Encouragingly, and in contrast to some prior indications from the literature [4], little evidence of stigmatizing or pejorative terms was observed; rather, we noted several empathic or humanizing adjectives reflecting strength and individual differences. Research with a variety of professional categories is needed, as this line of investigation would almost certainly prove fruitful to help direct our education, awareness, and advocacy efforts. In particular, targeting primary care professionals is important for early detection, given their likelihood of encountering an undiagnosed eating disorder along the initial pathway-to-care.
